# Supplementary material for: Hot spot prediction in protein-protein interactions by an ensemble system
Source: BMC Syst Biol. 2018 Dec 31;12(Suppl 9):132. doi: 10.1186/s12918-018-0665-8 (PMC6311905; doi:10.1186/s12918-018-0665-8)
Supplement: Supplementary file 2 — All relevant MATLAB code. A simple Matlab implement of our predictor. (ZIP 19,742 kb) [file 12918_2018_665_MOESM2_ESM.zip › Additional file 2/Read ASA/ASA website/dbMPIKT/4P5TA.html]

 NetSurfP - Protein Surface Accessibility and Secondary Structure Predictions

  

|  |  |  |
| --- | --- | --- |
|  |  | NetSurfP - Protein Surface Accessibility and Secondary Structure Predictions  Technical University of Denmark |

  


---

```
# For publication of results, please cite:
# A generic method for assignment of reliability scores applied to solvent accessibility predictions.
# Bent Petersen, Thomas Nordahl Petersen, Pernille Andersen, Morten Nielsen and Claus Lundegaard
# BMC Structural Biology 2009, 9:51 doi:10.1186/1472-6807-9-51
#
# Column 1: Class assignment - B for buried or E for Exposed - Threshold: 25% exposure, but not based on RSA
# Column 2: Amino acid
# Column 3: Sequence name
# Column 4: Amino acid number
# Column 5: Relative Surface Accessibility - RSA
# Column 6: Absolute Surface Accessibility
# Column 7: Z-fit score for RSA prediction
# Column 8: Probability for Alpha-Helix
# Column 9: Probability for Beta-strand
# Column 10: Probability for Coil
E M  4P5TA                  1    0.679 135.948  -0.317   0.003   0.003   0.994
B Q  4P5TA                  2    0.277  49.383  -0.793   0.021   0.279   0.699
E Q  4P5TA                  3    0.595 106.196   1.310   0.021   0.451   0.528
B V  4P5TA                  4    0.178  27.374  -0.064   0.021   0.451   0.528
E R  4P5TA                  5    0.518 118.714  -0.158   0.021   0.451   0.528
B Q  4P5TA                  6    0.232  41.507   0.180   0.020   0.205   0.775
E S  4P5TA                  7    0.424  49.716   0.075   0.018   0.047   0.935
E P  4P5TA                  8    0.510  72.326  -0.748   0.018   0.047   0.935
E Q  4P5TA                  9    0.553  98.677  -1.052   0.052   0.084   0.864
E S  4P5TA                 10    0.415  48.673  -1.177   0.021   0.451   0.528
B L  4P5TA                 11    0.196  35.943   0.041   0.021   0.756   0.223
E T  4P5TA                 12    0.442  61.333  -0.407   0.018   0.846   0.136
B V  4P5TA                 13    0.235  36.104   0.146   0.023   0.655   0.322
E W  4P5TA                 14    0.441 105.964   0.688   0.021   0.451   0.528
E E  4P5TA                 15    0.525  91.630   0.473   0.004   0.085   0.910
E G  4P5TA                 16    0.636  50.053  -1.085   0.005   0.015   0.979
E E  4P5TA                 17    0.492  85.952   0.418   0.004   0.138   0.858
E T  4P5TA                 18    0.563  78.116   1.296   0.003   0.718   0.279
B A  4P5TA                 19    0.079   8.662  -0.096   0.001   0.959   0.040
E I  4P5TA                 20    0.425  78.533   1.375   0.001   0.959   0.040
B L  4P5TA                 21    0.053   9.723   0.264   0.001   0.959   0.040
E N  4P5TA                 22    0.406  59.438   1.200   0.001   0.959   0.040
B C  4P5TA                 23    0.032   4.493   0.055   0.001   0.900   0.099
E S  4P5TA                 24    0.435  50.947   0.845   0.001   0.900   0.099
B Y  4P5TA                 25    0.237  50.647  -0.551   0.001   0.900   0.099
E E  4P5TA                 26    0.386  67.347  -0.928   0.003   0.718   0.279
E N  4P5TA                 27    0.460  67.417  -1.868   0.005   0.262   0.733
E S  4P5TA                 28    0.433  50.771  -2.248   0.018   0.088   0.893
E A  4P5TA                 29    0.489  53.888  -1.957   0.019   0.141   0.840
E F  4P5TA                 30    0.355  71.309  -1.402   0.019   0.141   0.840
B D  4P5TA                 31    0.282  40.651  -0.669   0.020   0.205   0.775
B Y  4P5TA                 32    0.123  26.306   0.325   0.023   0.655   0.322
B F  4P5TA                 33    0.029   5.720   0.377   0.018   0.846   0.136
B P  4P5TA                 34    0.074  10.543   0.388   0.018   0.846   0.136
B W  4P5TA                 35    0.028   6.830   0.463   0.018   0.846   0.136
B Y  4P5TA                 36    0.036   7.779   0.391   0.011   0.918   0.071
B Q  4P5TA                 37    0.142  25.325   1.004   0.018   0.846   0.136
B Q  4P5TA                 38    0.090  16.020   0.790   0.023   0.655   0.322
E F  4P5TA                 39    0.346  69.422   1.802   0.004   0.085   0.910
E P  4P5TA                 40    0.429  60.861   0.366   0.005   0.015   0.979
E G  4P5TA                 41    0.845  66.478  -1.824   0.003   0.003   0.994
E E  4P5TA                 42    0.431  75.366   0.348   0.018   0.019   0.964
E G  4P5TA                 43    0.397  31.236   0.200   0.018   0.047   0.935
B P  4P5TA                 44    0.074  10.501   0.386   0.021   0.279   0.699
B A  4P5TA                 45    0.242  26.635   0.689   0.018   0.846   0.136
B L  4P5TA                 46    0.084  15.362   0.293   0.011   0.918   0.071
B L  4P5TA                 47    0.021   3.863   1.255   0.006   0.962   0.032
B I  4P5TA                 48    0.022   4.126   0.731   0.001   0.959   0.040
B A  4P5TA                 49    0.080   8.849   0.256   0.000   0.983   0.017
B I  4P5TA                 50    0.077  14.264  -0.402   0.001   0.959   0.040
B R  4P5TA                 51    0.143  32.839  -0.190   0.021   0.756   0.223
B S  4P5TA                 52    0.205  24.003  -0.610   0.021   0.451   0.528
E V  4P5TA                 53    0.573  88.024  -1.512   0.019   0.141   0.840
E S  4P5TA                 54    0.484  56.690  -0.730   0.005   0.045   0.951
E D  4P5TA                 55    0.671  96.706  -1.667   0.018   0.047   0.935
E K  4P5TA                 56    0.705 144.936  -0.843   0.018   0.047   0.935
E K  4P5TA                 57    0.601 123.708  -0.751   0.018   0.088   0.893
E E  4P5TA                 58    0.409  71.435   0.002   0.018   0.088   0.893
E D  4P5TA                 59    0.674  97.123  -0.585   0.004   0.085   0.910
E G  4P5TA                 60    0.414  32.590  -1.360   0.004   0.085   0.910
B R  4P5TA                 61    0.218  49.968  -0.419   0.004   0.514   0.481
B F  4P5TA                 62    0.042   8.429  -0.216   0.002   0.816   0.182
B T  4P5TA                 63    0.207  28.655   0.132   0.001   0.900   0.099
B I  4P5TA                 64    0.090  16.650  -1.750   0.001   0.959   0.040
E F  4P5TA                 65    0.355  71.309   0.101   0.001   0.900   0.099
B F  4P5TA                 66    0.257  51.660  -0.742   0.001   0.900   0.099
E N  4P5TA                 67    0.448  65.543  -0.417   0.004   0.616   0.381
E K  4P5TA                 68    0.512 105.360  -0.946   0.004   0.197   0.799
E R  4P5TA                 69    0.601 137.606  -1.459   0.004   0.085   0.910
E E  4P5TA                 70    0.636 111.144  -0.653   0.004   0.138   0.858
E K  4P5TA                 71    0.365  75.060  -0.063   0.004   0.420   0.576
B K  4P5TA                 72    0.285  58.645   0.301   0.002   0.816   0.182
B L  4P5TA                 73    0.040   7.379  -0.320   0.001   0.959   0.040
B S  4P5TA                 74    0.218  25.526   0.603   0.001   0.959   0.040
B L  4P5TA                 75    0.029   5.310   0.401   0.001   0.959   0.040
E H  4P5TA                 76    0.274  49.822   0.736   0.001   0.959   0.040
B I  4P5TA                 77    0.067  12.377  -0.029   0.001   0.900   0.099
E T  4P5TA                 78    0.470  65.231   0.312   0.004   0.616   0.381
E D  4P5TA                 79    0.486  70.047  -0.406   0.021   0.279   0.699
B S  4P5TA                 80    0.148  17.346   0.105   0.004   0.197   0.799
E Q  4P5TA                 81    0.471  84.103   0.716   0.004   0.197   0.799
E P  4P5TA                 82    0.423  60.080  -0.428   0.020   0.205   0.775
E G  4P5TA                 83    0.500  39.319  -0.817   0.052   0.084   0.864
B D  4P5TA                 84    0.179  25.736  -0.965   0.056   0.142   0.802
B S  4P5TA                 85    0.213  24.940  -0.056   0.022   0.359   0.619
B A  4P5TA                 86    0.060   6.579  -0.634   0.021   0.756   0.223
B T  4P5TA                 87    0.157  21.831   0.904   0.018   0.846   0.136
B Y  4P5TA                 88    0.036   7.586   0.566   0.018   0.846   0.136
B F  4P5TA                 89    0.059  11.821   0.970   0.018   0.846   0.136
B C  4P5TA                 90    0.014   1.923   1.501   0.018   0.846   0.136
B A  4P5TA                 91    0.029   3.152   0.078   0.018   0.846   0.136
B A  4P5TA                 92    0.074   8.166  -0.216   0.021   0.756   0.223
B S  4P5TA                 93    0.133  15.552   0.283   0.004   0.514   0.481
E R  4P5TA                 94    0.396  90.798  -1.060   0.004   0.197   0.799
E D  4P5TA                 95    0.442  63.707  -2.023   0.005   0.045   0.951
E S  4P5TA                 96    0.421  49.318  -2.320   0.018   0.047   0.935
E G  4P5TA                 97    0.468  36.824  -1.957   0.018   0.047   0.935
E Q  4P5TA                 98    0.360  64.278  -0.761   0.018   0.047   0.935
E K  4P5TA                 99    0.331  68.148   0.312   0.004   0.197   0.799
B L  4P5TA                100    0.134  24.554   0.569   0.004   0.514   0.481
B V  4P5TA                101    0.055   8.407  -0.614   0.003   0.718   0.279
B F  4P5TA                102    0.043   8.670  -0.443   0.003   0.718   0.279
B G  4P5TA                103    0.064   5.053  -2.053   0.005   0.336   0.660
E Q  4P5TA                104    0.360  64.296   0.175   0.004   0.085   0.910
B G  4P5TA                105    0.230  18.132  -1.713   0.004   0.085   0.910
B T  4P5TA                106    0.109  15.049  -0.378   0.005   0.262   0.733
E I  4P5TA                107    0.372  68.839   0.780   0.002   0.816   0.182
B L  4P5TA                108    0.057  10.437   0.469   0.001   0.900   0.099
B K  4P5TA                109    0.227  46.776   0.515   0.001   0.900   0.099
B V  4P5TA                110    0.056   8.592  -0.361   0.001   0.900   0.099
E Y  4P5TA                111    0.342  73.107   0.568   0.002   0.816   0.182
E L  4P5TA                112    0.428  78.294  -1.587   0.004   0.514   0.481
E H  4P5TA                113    0.479  87.130  -0.936   0.019   0.141   0.840
E I  4P5TA                114    0.532  98.364  -1.547   0.018   0.088   0.893
E Q  4P5TA                115    0.540  96.426  -1.116   0.019   0.141   0.840
E N  4P5TA                116    0.521  76.333  -1.272   0.004   0.085   0.910
E P  4P5TA                117    0.362  51.396  -1.227   0.004   0.085   0.910
E D  4P5TA                118    0.574  82.771  -0.136   0.004   0.085   0.910
B P  4P5TA                119    0.109  15.467  -1.754   0.004   0.138   0.858
E A  4P5TA                120    0.360  39.639  -1.093   0.004   0.420   0.576
E V  4P5TA                121    0.329  50.598  -1.261   0.003   0.718   0.279
B Y  4P5TA                122    0.226  48.275  -0.497   0.023   0.655   0.322
E Q  4P5TA                123    0.382  68.189  -0.414   0.021   0.451   0.528
B L  4P5TA                124    0.119  21.752  -1.676   0.022   0.359   0.619
E R  4P5TA                125    0.394  90.203  -0.533   0.005   0.262   0.733
E D  4P5TA                126    0.497  71.618  -0.849   0.004   0.138   0.858
E S  4P5TA                127    0.379  44.454  -1.597   0.005   0.015   0.979
E K  4P5TA                128    0.737 151.601  -1.630   0.018   0.019   0.964
E S  4P5TA                129    0.620  72.617  -2.019   0.018   0.019   0.964
E S  4P5TA                130    0.552  64.659  -0.599   0.005   0.045   0.951
B D  4P5TA                131    0.252  36.299  -0.688   0.005   0.336   0.660
B K  4P5TA                132    0.236  48.483   0.357   0.003   0.718   0.279
B S  4P5TA                133    0.051   6.024   0.341   0.001   0.900   0.099
B V  4P5TA                134    0.039   6.071   0.118   0.001   0.900   0.099
B C  4P5TA                135    0.020   2.794   1.259   0.001   0.900   0.099
B L  4P5TA                136    0.055   9.997   1.059   0.001   0.900   0.099
B F  4P5TA                137    0.046   9.272   0.052   0.002   0.816   0.182
B T  4P5TA                138    0.226  31.277  -0.010   0.004   0.514   0.481
E D  4P5TA                139    0.475  68.419  -1.121   0.005   0.262   0.733
B F  4P5TA                140    0.160  32.152  -0.591   0.004   0.197   0.799
E D  4P5TA                141    0.310  44.700   0.292   0.005   0.262   0.733
B S  4P5TA                142    0.170  19.877  -1.750   0.004   0.138   0.858
E Q  4P5TA                143    0.568 101.373  -0.962   0.004   0.085   0.910
E T  4P5TA                144    0.406  56.312  -0.961   0.004   0.197   0.799
B N  4P5TA                145    0.073  10.673  -0.264   0.004   0.616   0.381
E V  4P5TA                146    0.324  49.737   0.058   0.002   0.816   0.182
B S  4P5TA                147    0.066   7.723  -0.572   0.001   0.900   0.099
E Q  4P5TA                148    0.269  48.043   0.717   0.001   0.959   0.040
B S  4P5TA                149    0.111  13.056  -0.057   0.001   0.900   0.099
E K  4P5TA                150    0.343  70.493   0.847   0.001   0.900   0.099
E D  4P5TA                151    0.451  64.975  -0.655   0.003   0.718   0.279
E S  4P5TA                152    0.665  77.915  -1.672   0.004   0.085   0.910
E D  4P5TA                153    0.662  95.452  -1.496   0.004   0.085   0.910
E V  4P5TA                154    0.568  87.348  -0.226   0.004   0.616   0.381
E Y  4P5TA                155    0.488 104.179  -0.119   0.003   0.718   0.279
E I  4P5TA                156    0.286  52.910  -0.051   0.003   0.718   0.279
E T  4P5TA                157    0.450  62.484  -0.736   0.004   0.616   0.381
E D  4P5TA                158    0.541  77.958  -1.264   0.005   0.262   0.733
E K  4P5TA                159    0.414  85.119  -1.252   0.005   0.336   0.660
B C  4P5TA                160    0.295  41.418  -1.532   0.002   0.816   0.182
E V  4P5TA                161    0.323  49.584  -0.117   0.001   0.900   0.099
E L  4P5TA                162    0.350  64.030   0.442   0.001   0.900   0.099
E D  4P5TA                163    0.287  41.357  -0.268   0.021   0.756   0.223
E M  4P5TA                164    0.337  67.434  -0.072   0.004   0.616   0.381
E R  4P5TA                165    0.564 129.133  -0.406   0.004   0.420   0.576
E S  4P5TA                166    0.568  66.534  -1.418   0.004   0.197   0.799
E M  4P5TA                167    0.567 113.457  -1.838   0.004   0.138   0.858
E D  4P5TA                168    0.601  86.633  -1.853   0.004   0.197   0.799
E F  4P5TA                169    0.302  60.591  -1.321   0.004   0.616   0.381
B K  4P5TA                170    0.187  38.404  -0.738   0.002   0.816   0.182
B S  4P5TA                171    0.144  16.865  -0.839   0.001   0.900   0.099
B N  4P5TA                172    0.102  14.904   0.022   0.001   0.900   0.099
B S  4P5TA                173    0.128  14.990  -0.293   0.001   0.900   0.099
B A  4P5TA                174    0.070   7.714  -1.540   0.018   0.846   0.136
B V  4P5TA                175    0.157  24.069  -1.717   0.018   0.846   0.136
B A  4P5TA                176    0.245  26.988  -0.174   0.002   0.816   0.182
B W  4P5TA                177    0.127  30.592  -1.374   0.004   0.514   0.481
E S  4P5TA                178    0.523  61.307   0.481   0.004   0.138   0.858
E N  4P5TA                179    0.677  99.098   0.545   0.005   0.015   0.979
E K  4P5TA                180    0.757 155.653  -0.441   0.005   0.015   0.979
E S  4P5TA                181    0.633  74.234   0.324   0.004   0.138   0.858
E D  4P5TA                182    0.537  77.324   1.498   0.003   0.718   0.279
B F  4P5TA                183    0.099  19.889   1.165   0.001   0.900   0.099
E A  4P5TA                184    0.429  47.298   1.797   0.001   0.959   0.040
B C  4P5TA                185    0.032   4.479  -0.751   0.001   0.959   0.040
E A  4P5TA                186    0.400  44.091   1.575   0.001   0.900   0.099
B N  4P5TA                187    0.101  14.845   0.131   0.011   0.918   0.071
E A  4P5TA                188    0.503  55.420   0.894   0.001   0.900   0.099
B F  4P5TA                189    0.201  40.301   0.114   0.003   0.718   0.279
E N  4P5TA                190    0.661  96.829   0.220   0.004   0.085   0.910
E N  4P5TA                191    0.711 104.105  -1.190   0.005   0.015   0.979
E S  4P5TA                192    0.650  76.227  -0.792   0.004   0.197   0.799
E I  4P5TA                193    0.446  82.510  -0.511   0.004   0.420   0.576
E I  4P5TA                194    0.406  75.147   0.340   0.004   0.514   0.481
E P  4P5TA                195    0.428  60.733  -0.033   0.022   0.552   0.426
E E  4P5TA                196    0.515  90.040  -1.190   0.021   0.451   0.528
E D  4P5TA                197    0.572  82.353  -0.402   0.021   0.451   0.528
E T  4P5TA                198    0.561  77.811   0.703   0.021   0.451   0.528
E F  4P5TA                199    0.291  58.464  -0.463   0.022   0.359   0.619
E F  4P5TA                200    0.437  87.686  -1.078   0.005   0.336   0.660
E P  4P5TA                201    0.583  82.714  -0.106   0.004   0.197   0.799
E S  4P5TA                202    0.412  48.333  -0.098   0.004   0.085   0.910
E P  4P5TA                203    0.653  92.632  -1.080   0.019   0.141   0.840
E E  4P5TA                204    0.629 109.799  -0.294   0.018   0.088   0.893
B S  4P5TA                205    0.246  28.796  -2.167   0.018   0.047   0.935
E S  4P5TA                206    0.751  88.017  -2.373   0.003   0.003   0.994

Explain the output.  Go back.
```

---
